# Perforation of the Appendix Vermiformis

**Published:** 1887-09

**Authors:** 


### PERFORATION OF THE APPENDIX VERMIFORMIS.

Dr. J. McF. Gaston, our collaborator, presents the following summary of a paper read bv him at the late meeting of the American Medical Association:

The practical deductions from this inquiry in regard to the concomitants of perforation of the appendix vermiformis may be included under the following heads:

1. The primary disorder is dependent upon a local irritant, either mechanical, chemical, or vital, inducing ulceration and disintegration at some point in its walls.

2. The modification in the tissues of adjacent parts depends upon the presence of a toxic exudation from its cavity, that ultimately leads to disorganization of structure.

3. Extension of the degenerating process depends upon the permeation of the structures with the fæcal matter, but may result from suppuration. or the automatic propagation of inflammation from one part to another.

4. Agglutinations between the layers of peritoneum may shut in purulent accumulation and thus limit the inflammatory action to a circumscribed area so as to assume the nature of an abscess in that locality.

5. General peritonitis may be accompanied with extensive adhesions of the adjacent serous membrane and followed by vital prostration and collapse, calling for the knife.

6. Septicæmia may occur from absorption of septic matter, independent of suppuration, and associated with a low form of fever, which ought to be treated with antiseptics and irrigations of the abdominal cavity with hot water.

7. When there are sufficient indications of perforation in the general symptoms, with pain and tenderness on pressure over the cæcal region, without signs of fluctuation, an exploratory puncture below the ileo-cæcal junction is warranted.

8. If there are reasonable grounds to believe that pus is present, or there is fæcal matter, whether from the perforation of the cæcum or appendix, a free incision above Poupart's ligament should be carried down to these parts and drainage kept up afterwards.

9. In perforations of the appendix associated with general peritonitis, an incision in the linea alba offers the best prospect of reaching all the parts involved, and should be accompanied by thorough cleansing of the abdominal cavity, and especially the ileo cæcal region.

10 The most efficient means of closing an opening in the cæcum is by Lembert's suture, while one in the appendix demands excision and ligation.

11. When perforation is suspectd, washing out the abdomen by the use of a syringe and two tubes will assist in the diagnosis and treatment

12. An early operation, with a doubtful diagnosis of perforation of the appendix, lessens the liability for a confirmation of it by a necropsy, and, hence, no time should be lost in awaiting developments.

